# How cancer arises: Genetics releases, plasticity creates, genetics stabilizes

**DOI:** 10.1073/pnas.2505377122

**Published:** 2025-07-31

**Authors:** Steven A. Frank

**Affiliations:** ^a^Department of Ecology and Evolutionary Biology, University of California, Irvine, CA 92697-2525

**Keywords:** developmental plasticity, cancer evolution, cell state, single-cell technology

## Abstract

Cancer is the origin of a novel tissue that attracts resources, spreads beyond boundaries, avoids normal controls, and escapes immunity. How does a novel tissue arise? The puzzle is that two seemingly different processes appear to be the primary driving force. On the one hand, overwhelming evidence links (epi)genetic driver mutations to the origin and progression of tumors. Common oncogenic mutations such as *KRAS* accelerate cell division, and common knockouts of tumor suppressors such as *TP53* abrogate cell death or checks on cell division. On the other hand, cancerous tissues create complex traits that require intricate changes in cells and multiple interactions between different cell types. Such novelty often arises by hijacking the developmental plasticity that normally creates the diverse cells and tissues of our bodies from a single original zygotic cell. How can we reconcile the simple genetic changes in carcinogenesis with the complex developmental plasticity that creates novel tissues? This perspective advocates a new model. (Epi)genetic mutations release developmental plasticity. That developmental plasticity creates novel cellular interactions and complex tissues. Initially, novel traits created by developmental plasticity may not be stably heritable, thus subsequent (epi)genetic changes must stabilize the phenotypic novelty. Recent studies show how classic oncogenic and tumor suppressor driver mutations, such as *KRAS* and *TP53*, may primarily act in early carcinogenesis as broad releasers of developmental plasticity rather than as stimulators of cell division or knockout of limitations on cellular clonal expansion. In the new model, genetics releases, plasticity creates, and genetics stabilizes.

Cancer is often viewed as arising from driver mutations that increase cell division and decrease cell death. Normally, cells follow a tightly regulated cycle, receiving signals to limit cell division and to self-destruct when damaged beyond repair. In cancer, mutations disrupt these regulatory processes, leading to uncontrolled growth (1–3).

However, recent single-cell studies show that classic driver mutants, such as *KRAS* and *TP53*, also unlock cells from their terminally differentiated developmental state. Freed from normal constraints, cells wander stochastically over various dedifferentiated cellular programs (4–6).

The return to the highly plastic tissue-building states of normal development releases the potential to create novel tissues that attract resources, spread beyond boundaries, avoid normal controls, and escape immunity. Subsequent (epi)genetic mutations may stabilize the novel phenotypes, which initially arise by developmental plasticity.

This theory for the plasticity-led creation of cancer parallels an emerging perspective in evolutionary biology. In evolution, novel traits may first arise by developmental plasticity rather than by new mutations. That initial variation by plasticity may then subsequently be modulated and stabilized by genetic change (7, 8).

In prior articles, we argued that these new ideas may substantially change how we understand the origin and spread of the novel traits that drive cancer progression and the resistance to treatment (9, 10). The existing data remain suggestive rather than conclusive. However, the supporting observations from the relatively new single-cell studies of cancer deserve wide attention.

This perspective summarizes where we are in the history of this topic, some compelling recent studies, and my opinion for the likely future changes in how we understand the origins of cancers.

I also link the evolutionary processes that drive the origin and spread of tumors to the new ideas from evolutionary theory about developmental plasticity. That synergism between our understanding of cancer evolution and general evolutionary processes suggests an opportunity for an enhanced conceptual foundation, making new predictions that advance understanding in both domains.

## Brief History

Much evidence supports the genetic driver view (11, 12). Certain mutations typically recur in tumors. *KRAS* mutations associate with greater cell division (13). *TP53* mutations associate with knockout of programmed cell death (14). Cells that divide faster and fail to die create an expanding tumor.

However, for many years, a disquiet undercurrent flowed through the field (15, 16). An individual may have many of the key cancer-driving mutations in their pancreas but no overt signs of tumor (4). Other organs can also be mutated with characteristic cancer drivers without disease (17).

Certainly, the mutations play an important role in tumors. But perhaps they are not sufficient, not the sole driving cause. Critics often pointed to the broad mixture of different kinds of cells in a tumor.

A lung tumor includes immune cells and connective tissue cells. Usually, the driver mutations are in the lung cells but not in the other cell types. In other tumors, the key genetic changes are also in the primary cell type but typically not in the various partner cells. Maybe the cellular collaboration creates a local microenvironment that a tumor needs to get started, to survive attack by host immunity, to break through tissue boundaries, and to spread aggressively (15, 16, 18).

The tumor microenvironment idea was compelling but hard to study or to use as the basis for treatment. By contrast, it became easy to link certain genetic mutations with the progression of particular tumors. The mutations also identified genes and cellular functions to treat, with some success.

Ideas about plasticity and developmental dedifferentiation also complemented the genetic aspect (19–24). Epithelial cells often revert to a developmentally more primitive and highly plastic mesenchymal cell type during the later stages of cancer progression and metastasis. Mesenchyme can express a wide variety of phenotypes, transit into various cell states, and promote changes that advance aggressive tumors.

However, the plasticity associated with the epithelial to mesenchymal transition (EMT) in late-stage cancers was not typically emphasized as a driver for the origin of tumors. Instead, the common view for the origin of tumors focused on new mutations that drive the early stages of clonal expansion in cellular lineages. So, the theory that plasticity might be a primary driver early in cancer (9) was mostly ignored. Then, a new method revealed previously hidden roles for plasticity in the early stages of tumor initiation (10, 25–28).

## A New Tool

Single-cell technology can measure the state of individual cells. Measurements include transcriptomic, epigenomic, and proteomic readouts with genome-wide resolution. By tracking what happens in each cell and using barcodes to trace cellular lineages, one can directly study the genetic and physiological attributes that cause changes in cellular populations (29). This technique provides an ideal tool to follow the initiation and progression of tumors (30). Previously, one had to aggregate millions or billions of cells to measure the average composition of a tissue.

Imagine, for example, that one could only measure diabetes as the average incidence over an entire country. Insight could only come by comparing countries. Then, new methods measured diabetes in each individual. One could then compare individual characteristics with disease onset, tracking the origin and progression of changes within each individual. It is like using the first microscope, seeing previously hidden causes for the first time.

The single-cell revolution will alter our understanding of how cancer arises. Already, there is enough evidence to suggest a new perspective. Instead of genetic mutations driving the early steps of cellular change in cancer, those first mutations may primarily release cellular plasticity. That plasticity may trigger the sort of widespread cellular interactions that drive the changes of normal development (10).

## The Developmental Origin of a Novel Tissue

As the tumor microenvironment literature emphasized (15, 16), cancer is ultimately the origin of a novel tissue. It is a tissue created by natural selection acting within the body, designed to attract resources, spread beyond boundaries, avoid normal controls, and escape immunity. How does a novel tissue arise?

A novel tissue arises in the same way that bodies are built from nothing, by development. Cancer is normal development spun out of control. It is the great plasticity and power of development, without the overarching controls that guide normal development toward an integrated adult form.

Whenever a newly developed kind of tissue acquires the ability to survive, grow, and resist control, there is nothing to stop it. That may be why normal epithelial cells are often terminally differentiated into a restricted cellular program. And it may be why wound healing, closely related to powerfully plastic tissue remodeling, is so tightly regulated and, when dysregulated, so often associates with cancer (31, 32).

## Developmental Plasticity as a Primary Driver

The current genetic-driver perspective has been highly successful. Altering a successful perspective demands overwhelming evidence. So far, five lines of evidence suggest how this change might eventually happen.

### Plasticity in Late-Stage Tumors.

Epithelial cells in tumors often reverse normal development, taking on the dedifferentiated mesenchymal state (19). Mesenchyme expresses great developmental plasticity, with the ability to remodel tissues. The epithelial to mesenchymal transition has for many years been a widely known part of the current perspective, primarily causing changes limited to late-stage tumors and metastatic spread. So, although late-stage plasticity has become a commonly discussed hallmark of cancer (33), the genetic driver perspective continues to dominate ideas about how tumors get started, in spite of several attempts to emphasize plasticity as a primary driver (4–6, 9, 10, 25, 26).

The limited role ascribed to plasticity as a primary driver continues because of the widely observed link between classic cancer genetic mutations and the early stages of progression. However, new single-cell studies may resolve the apparent discord between the genetic evidence and the importance of plasticity, opening the way for a broader understanding of plasticity’s essential role.

### Classic Driver Mutations Release Early Plasticity.

Recent studies elevate developmental plasticity to the primary driving role in early-stage tumors. In mouse lung adenocarcinoma, an initial *Kras* mutation abrogates strict terminal differentiation of lung epithelium. The cell states wander stochastically over a landscape of altered cellular types. A further *Tp53* mutation triggers broader dedifferentiation into a variety of cell types, some of which include parts of the developmental programs of trophoblasts, chondroblasts, and kidney tubular epithelium (6).

Here is the key point. It seems that the classic *Kras* oncogene driver mutation and *Tp53* tumor suppressor driver mutation most importantly trigger dedifferentiation and developmental plasticity. Those mutations may also cause some acceleration of cell division and knockout of normal cell death. But those effects on cell birth and death seem to be secondary or complementary to the primary effect on developmental plasticity.

The developmental plasticity initiates the creation of the novel tissue characteristics that define an aggressive tumor. Access to the developmental programs of trophoblast placental remodeling, chondroblast structural traits, and kidney epithelium immune modulation likely plays an important role in building a novel cancer tissue. The classic genetic mutations come first but act as releasers of developmental plasticity rather than as direct drivers of cellular growth and survival.

Another recent mouse study of lung cancer evolution also identifies *Kras* and *Tp53* mutations as early releasers of developmental plasticity (5). The cells that line the lung alveolar surface include the common thin and flat AT1 cells that facilitate gas exchange. The lung epithelium also includes rarer AT2 cells that produce surfactant and act as stem cells to repair injury. During normal development and in wound healing, the relatively dedifferentiated AT2 cells produce the terminally differentiated AT1 cells.

In development, TP53 triggers AT2 to AT1 differentiation via a cell type that resembles an intermediate in alveolar injury repair. In a mouse model with a *Kras* mutation in AT2 cells, the subsequent knockout of *Tp53* produced an excess of the intermediate repair-like state (5). This highly plastic cell type is an important stage in this mouse model’s progression toward lung cancer.

These lung cancer studies emphasize that classic early driver mutations release developmental plasticity. Cancer arises as the origin of a novel tissue by normal developmental processes spun out of control. This perspective contrasts with the currently dominant view by which the primary early driver mutations increase cell division and abrogate cell death. In other words, the new perspective changes the focus from how a cell lineage expands to how a novel, complex, and aggressive tissue arises.

The point is not that developmental plasticity excludes increased self-renewal and reduced cell death. Release of primordial developmental programs may in fact associate with greater cellular birth and reduced cellular death. However, the primary issue concerns how traits arise that overcome the barriers to tumor formation and spread. The important evidence will therefore have to do with the origin of processes such as immune escape, angiogenesis, the breakdown of physical barriers, and so on. Do such traits first appear by the release of latent developmental programs and their recombination?

### Further Evidence for Plasticity as the Early Driver.

A recent mouse study of pancreatic cancer progression supports the view that plasticity is the key early driver for the origin of tumors (4). *Kras* mutations happen early but, by themselves, have little direct effect. Instead, those mutations trigger cell-state plasticity. That cellular plasticity causes different cell states to express unique sets of ligands and receptors involved in cell–cell signaling between epithelial and immune cells.

The phenotypic variability in cellular interactions may provide the basis for creating a novel tumor microenvironment. Experimental studies identified one example, in which some Kras-induced states of pancreatic epithelial cells express the immune signaling molecule IL-33, which triggers T cells to respond by secreting IL-4, to which some of the Kras-mutant epithelial cells apparently respond via an expressed receptor for IL-4.

This kind of positive feedback may be important in the early stages of a pancreatic tumor microenvironment, triggering the initial steps in creating the novel cancerous tissue that resists immune control.

### Developmental Dedifferentiation and Drug Resistance.

Studies link cellular plasticity and dedifferentiation to drug resistance (9, 10, 34, 35). In melanoma, cells fluctuate stochastically between terminally differentiated melanocytes and their developmental precursors, neural crest cells (36, 37). The neural crest cells resist treatment, expressing the dedifferentiated properties of stress resistance, migratory tendency, and plasticity of cellular traits.

Upon treatment, subsequent genetic mutations stabilize the previously fluctuating cell states toward the resistant neural crest form. Here, mutations lock in advantageous expression among plasticity’s phenotypic range. This process illustrates how evolutionary change can happen by two steps. First, advantageous traits initially arise by nonheritable phenotypic variety. Second, the traits are stabilized by subsequent (epi)genetic change.

The initial phenotypic variety may simply be from stochastic fluctuations (38, 39). In this case of melanoma resistance, it appears that random fluctuations in the cellular abundance of a transcription factor shift cell state between melanocytes and neural crest-like precursors. In other cases, the first phenotypes that provide resistance or steps in carcinogenesis may arise by cellular plasticity, the responsiveness of cells to the environment (9, 10, 27, 40).

For example, in the evolution of melanoma resistance to immunotherapy, the first steps may arise by inflammation-induced cellular dedifferentiation (36, 41). Tissue culture organoids sometimes gain their first steps toward resistance to drugs by a cellular stress response that suppresses MYC, a master regulator of biosynthesis and metabolism (42). Suppressing MYC can cause cells to switch to an embryonic diapause state that stops cell division and resists many drugs.

Although an initial cell state shift may first happen stochastically, as in the first melanoma example, the new cell state often expresses greater developmental plasticity. The greater developmental plasticity of the new cell state may be the primary driver of subsequent evolutionary change in resistance or carcinogenesis.

### Plasticity as the Primary Evolutionary Driver of Biological Novelty.

Finally, the idea that plasticity drives the origin of novelty synergizes with recent theories in evolution (7, 8). Traditionally, new traits in evolution are thought to arise by genetic mutation and then, if advantageous, to spread and take over the population. Alternatively, recent compelling arguments put phenotypic plasticity first. When faced with a new environmental challenge, the normal flexibility of organisms often leads to nongenetic changes that partially meet the challenge.

A good partial solution expressed by a developmentally plastic program can be modulated and stabilized by subsequent genetic or epigenetic changes. In this scenario, the origin of a new trait first happens by altering the expression of existing developmental programs. Then the genetic changes follow. An increasing number of studies support the idea that phenotypic plasticity can lead in the creation of novelty (43, 44) (Box 1).

Box 1. Two examples of plasticity-led evolution in nature

Plasticity-led evolution occurs when an environmental trigger induces latent developmental potential, generating a novel, integrated phenotype in a single step. The new trait is later refined and stabilized by selection of genetic variants.

**Ant supersoldiers (*Pheidole*)—**A spike of juvenile hormone in well-fed larvae triggers an ancestral, normally silent developmental module that produces workers with enlarged heads and mandibles, modified musculature and nervous system, and a characteristic defensive behavior that protects their colonies against attack. This particular response to juvenile hormone occurs in most or all of the genus. Natural selection has stabilized the initially plastic response in at least two independent lineages, which now express supersoldiers at elevated frequency (45–47).

**Carnivore morph in spade-foot toads (*Spea*)—**Abundant fairy shrimp food triggers tadpoles to develop into a carnivorous phenotype. The plastic developmental program coordinately enlarges the jaw muscles, hardens the beak, shortens the gut, and accelerates development. These carnivores grow faster than omnivores, more successfully escaping evaporating ponds. Some populations of *Spea bombifrons* have secondarily become obligately carnivorous independently of diet, illustrating the subsequent stabilization of the inducible ancestral form (48).

In both cases, a single trigger induces a well-integrated set of traits. Subsequent selection tunes the threshold and degree of expression, transforming an initially plastic developmental program into a partially or completely stable genetic program. In other cases, induction of developmental plasticity may create novel phenotypes that are initially not so well formed, providing the raw material for subsequent refinement into complex adaptations.

In both cancer and the history of life, complex novel traits sometimes arise. That leads to one of the great puzzles in biology. Given that natural selection culls weakly performing intermediates, what sequence of steps produces a complex novel trait?

West-Eberhard (7, 8) emphasized developmental recombination. A new phenotype is created when different developmental programs are combined in a new way. In a famous example, a goat was born with nonfunctioning front legs (49, 50). By walking and running on its back legs from birth, it developed “remarkable changes in muscle and bone, including striking changes in the bones of the hind legs; [altered] leg muscles, including a greatly thickened and elongated gluteal tongue and an innovative arrangement of small tendons, a modified shape of the thoracic skeleton, and extensive modifications of the pelvis” (8).

The novelty of walking on two legs appeared all at once. That complex trait arose from the recombination of different developmentally plastic programs for muscles, bones, connective tissue, and behavior. Those different programs were integrated by an overarching developmental robustness that directed the parts into a well-functioning whole.

In general, plasticity is often an evolutionary adaptation designed to alter phenotype in a beneficial way in response to a changed environment. Within cells, many transcription factors act as switches to turn on complex, multicomponent traits. By contrast, random mutations or epigenetic modifications rarely create broad, well-integrated phenotypic changes in one step. For these reasons, plasticity may often be the primary source of advantageous novelty.

Such extreme novelty in one step is not necessarily an essential part of carcinogenesis and drug resistance. However, the interesting case discussed earlier of mouse lung adenocarcinoma may be an example of novelty by developmental recombination.

In that study, 12 different cell types recurred during tumor progression in different animals (6). Most of the cellular diversity arose by reversing the normal lines of cellular differentiation during development. Early forms of cellular diversity included alternative lung epithelial states. Then, several cell states arose that were similar to primordial gut cells. In the middle of the expanding sequence of cellular phenotypic diversity, a highly plastic state (mixed program) arose as a transitional form.

The authors summarized their main observations by noting that “the highly mixed program displayed features of drastically different cell types, ranging from trophoblast stem cells to chondroblasts and kidney tubular epithelium…. [ellipsis then space then period]” That quote perfectly describes developmental recombination, a new phenotype by the mixture of different developmental programs.

The authors arrived at their interpretation empirically, from what they observed in their experiments. They emphasized that the recombined developmental program has the potential to create a novel, complex, and aggressive cancer tissue by mixing the aggressive growth properties and immune evasion of placental tissue derived from trophoblasts (51) with the connective tissue properties of chondroblasts and the immune modulatory properties of kidney tubular epithelium. This independent empirical derivation of the concept of developmental recombination lends support to the broad theoretical framework of plasticity-led novelty in the evolutionary origin of complex novel traits.

The essential point is that functional novelty is more likely to arise from developmental plasticity than from random (epi)genetic change. Although the initial phenotype produced by plasticity may not be heritable, secondary heritable stabilization of advantageous novelty may evolve relatively easily by subsequent (epi)genetic modification of regulatory controls.

These principles apply to cancer and to general aspects of evolutionary novelty. Thus, the study of cancer will teach us more about how evolution works, and progress in evolutionary biology will help to understand the processes that drive carcinogenesis and resistance.

## Linking (Epi)genetics and Plasticity

Of course, the traditional path of mutations coming first does occur. But for true novelty, in which there is a major change of phenotype in response to extreme challenge, plasticity-led evolution is a plausible and perhaps ultimately dominant pathway.

The case for plasticity as the primary leading cause in the creation of biological novelty has not been proved in tumors or in evolution. However, the trends in cancer research revealed by single-cell technology and in recent evolutionary studies suggest that a new perspective may be coming.

For cancer, the new storyline is that genetic and epigenetic changes may initially trigger the release of cell-state variability and developmental plasticity. Interestingly, classic cancer mutations such as *KRAS* and *TP53* may be most important as releasers of developmental plasticity rather than as an oncogene or a knockout of a tumor suppressor. In a few recent studies, the released plasticity plays the primary creative role. Subsequent genetic and epigenetic changes stabilize the newly created cellular interactions of the aggressive tissue.

**Fig. 1.**
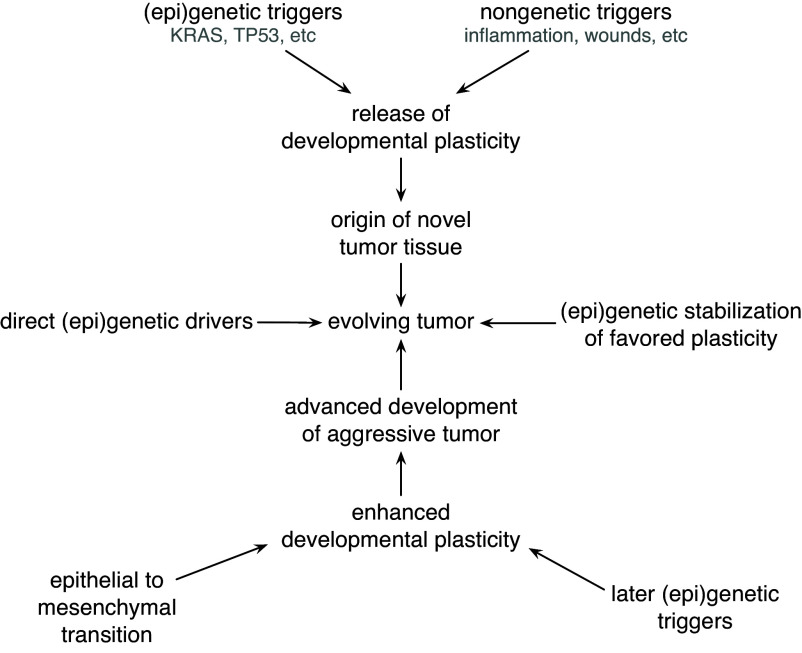
Interactions between plasticity and (epi)genetics in the origin and progression of tumors. The upper-right pathway emphasizes that, in some cases, environmental induction of plasticity by inflammation, wounding, or stress may be sufficient to release the initial driving plasticity or may be a complementary cause to initial mutations that release plasticity.

In this narrative, genetic and epigenetic instabilities accelerate cancer by increasing the release of plasticity or by enhancing the rate at which advantageous phenotypic novelty becomes heritably stabilized. The idea is that, often, genetics triggers, plasticity creates, and genetics stabilizes (Fig. 1).

Certainly, the traditional mutation and selection processes also play important roles. Here, we are striving for a more balanced view of the complementary roles of the cast rather than evicting formerly dominant players. New studies will clarify the relative importance of the alternative processes.

Inevitably, different tissues and different types of cancer will vary in the relative importance of traditional mutation–selection processes versus plasticity. For example, it is not surprising in retrospect that melanoma is a particularly aggressive cancer given the derivation of melanocytes from the highly migratory and exceptionally plastic neural crest cell state (52).

It seems that the relatively easy dedifferentiation of melanocytes back to the neural crest state explains why melanoma is so aggressive and another skin cancer, basal cell carcinoma, is not. Basal cells derive developmentally from surface ectoderm, which is likely not as plastic or migratory as neural crest cells (52).

Eventually, we may understand many of the differences between tumor types by the cell states that are most easily reachable from the normal state of a tissue (53). The developmental hierarchy provides clues but, as in the mouse lung adenocarcinoma example, a particular cell type released from its terminally differentiated state may vary over several different cell types following mutations that release terminal differentiation, such as *KRAS* and *TP53* appear to do in certain tissues.

Also, it will be important to understand how various mutations release particular aspects of developmental plasticity. For example, mutation of what seem to be broadly acting oncogenes or tumor suppressors often have surprising tissue specificity in their effects on cancer (54). That puzzle may be solved if such mutations are instead acting primarily as releasers of developmental plasticity in particular tissues. Better understanding of the associations between mutations and developmental plasticity will lead to deeper understanding of how particular germline and somatic mutations influence particular types of cancer.

## Data Availability

There are no data underlying this work.
